# Characteristics of individuals receiving disability benefits in the Netherlands and predictors of leaving the disability benefit scheme: a retrospective cohort study with five-year follow-up

**DOI:** 10.1186/s12889-018-5068-7

**Published:** 2018-01-18

**Authors:** Ilse Louwerse, Maaike A. Huysmans, H. Jolanda van Rijssen, Allard J. van der Beek, Johannes R. Anema

**Affiliations:** 1 Department of Public and Occupational Health, Amsterdam Public Health Research Institute, VU University Medical Center, Van der Boechorststraat 7, 1081 BT Amsterdam, The Netherlands; 2 Dutch Institute of Employee Benefit Schemes (UWV), Amsterdam, The Netherlands; 3 Research Center for Insurance Medicine, AMC-UMCG-VUmc, Amsterdam, The Netherlands

**Keywords:** Disability benefit entitlement, Diagnosis, Mental disorders, Comorbidity, Continuing eligibility

## Abstract

**Background:**

Today, work disability is one of the greatest social and labour market challenges for policy makers in most OECD countries, where on average, about 6% of the working-age population relies on disability benefits. Understanding of factors associated with long-term work disability may be helpful to identify groups of individuals at risk for disability benefit entitlement or continuing eligibility, and to develop effective interventions for these groups. The purpose of this study is to provide insight into the main diagnoses of workers who qualify for disability benefits and how these diagnoses differ in age, gender and education. Using a five-year follow-up, we examined the duration of disability benefits and how durations differ among individuals with various characteristics.

**Methods:**

We performed a cohort study of 31,733 individuals receiving disability benefits from the Dutch Social Security Institute (SSI) with a five-year follow-up. Data were collected from SSI databases. Information about disorders was assessed by an insurance physician upon benefit application. These data were used to test for significant relationships among socio-demographics, main diagnoses and comorbidity, and disability benefit entitlement and continuing eligibility.

**Results:**

Mental disorders were the most frequent diagnosis for individuals claiming work disability. Diagnoses differed among age groups and education categories. Mental disorders were the main diagnosis for work disability for younger and more highly educated individuals, and physical disorders (generally musculoskeletal, cardiovascular and cancer) were the main diagnosis for older and less educated individuals. In 82% of the claims, the duration of disability benefit was five years or more after approval. Outflow was lowest for individuals with (multiple) mental disorders and those with comorbidity of mental and physical disorders, and highest for individuals with (multiple) physical disorders.

**Conclusions:**

The main diagnosis for persons entitled to disability benefits was mental health problems, especially for young women. In a five-year follow-up, claim duration for disability benefits was long lasting for most claimants.

## Background

Today, work disability is one of the greatest social and labour market challenges for policy makers in most OECD countries [1]. On average, about 6% of the working-age population relies on disability benefits. Public spending on these benefits has become a serious burden. In the Netherlands, spending on disability benefits has risen to 4% of the gross domestic product. Moreover, once a disability benefit has been approved, the probability of returning to work is low [1]. Long-term unemployment or occupational inactivity is bad for an individual’s health, especially those with mental health conditions, and returning to work is generally associated with health improvement [2–4]. Thus, prevention of work disability and support for returning to work are in the interest of individuals and society as a whole.

Dutch social security legislation allows workers to apply for a disability benefit after two years of sick leave (see Table 1) [5]. Disability benefits can be approved for a disease or handicap due to either social (i.e. non-occupational) or occupational causes.

**Table 1:** Disability benefits in the Netherlands

In the Netherlands, disability benefits are assessed by the Dutch Social Security Institute (SSI). After two years of sick leave, individuals can apply for a disability benefit under the Dutch Work and Income Act (WIA). A medical disability assessment (i.e. diagnoses and functional abilities) is conducted by an insurance physician (IP) who is employed by the SSI. Depending on the functional abilities listed in the IP’s report, there may also be an assessment by a labour expert who calculates the loss of former wages. A disability benefit is granted if loss of income exceeds 35% of former wages. Disability benefits can be approved for a disease or handicap due to either social (i.e. non-occupational) or occupational causes.
Certain circumstances may change a person’s continuing eligibility for disability benefits. A disability benefit ends if the SSI IP determines that the medical condition has improved substantially and the labour expert calculates that loss of income is less than 35% of former wages. Other main causes are retirement and death.

Return-to-work patterns and the effectiveness of interventions aimed at a return to work differ across individuals and their specific characteristics and health conditions [6, 7]. In Sweden, the majority of psychiatric outpatients with depression were female, less than 44 years, and had completed more than 9 years of compulsory education [8]. Moreover, the duration of mental health-related disability was influenced by socio-demographic factors such as age and education, and clinical factors such as comorbidity [9, 10]. A Dutch study among cancer survivors following 2 years of sick leave concluded that among other factors, higher education, physical limitations and low self-reported work ability were associated with an increased risk for work disability [11]. For other physical disorders, such as lower back pain and major limb trauma, older age, low education level and smoking were significant predictors for long-term work disability [12, 13].

Whereas most studies about individuals at risk for long-term disability benefits and factors affecting return to work focus on a specific diagnosis, an overview of all diagnoses is missing. Therefore, in the present study we included all individuals who had been granted a long-term disability benefit in the Netherlands. An overview of individual characteristics provides insight into the range of diagnoses and how they differ among age groups, gender and education. This approach can help to target specific at-risk groups and identify effective interventions to prevent long-term work disability. Therefore, the aim of this study was twofold. The first aim was to identify the most important diagnoses for which individuals claim an inability to work, and to examine how diagnoses differ among age groups, gender and educational levels. Second, using a five-year follow-up, we aimed to determine the duration of the disability benefit and how durations differed among individuals with various characteristics.

## Methods

### Study population

The study cohort included 31,733 subjects who had been granted a WIA disability benefit by the SSI between July 2010 and June 2011 after a medical disability assessment by an IP. Subjects in the study sample were assessed as having a full and permanent work disability, non-permanent but full work disability, or permanent and partial work disability. Individuals in the latter group had some work capacity and were possibly enrolled in a (part-time) job. Adults disabled since childhood were not included in the study sample since in the Netherlands they are not entitled to a WIA disability benefit (instead they can apply for a Disablement Assistance Act for Handicapped Young Persons disability benefit when they turn 18).

### Socio-demographics

Socio-demographic data including gender, age and education are registered in the SSI database upon application for benefits. For further analysis, age was categorized into four groups: < 35, 35–44, 45–54 and 55+ years. Three education levels were defined based on the highest level of education completed; low (primary school, lower vocational education, lower secondary school), secondary (intermediate vocational education, upper secondary school), and high (upper vocational education, university). The educational level is usually registered during the labour expert’s assessment. Since this assessment is not necessary when the IP assesses full and permanent work disability, the education level was missing for 4036 individuals in our study sample. We excluded these individuals from the analyses concerning the education level as we could not deduce any information about their educational level and were therefore not able to use the results.

### Disorders

When applying for a disability benefit, the assessment of diagnoses and functional abilities is done by an IP who is employed by the SSI. The IP lists disorders according to the Dutch Classification of Occupational Health and Social Insurance (CAS). The CAS is based on the International Statistical Classification of Disease and Related Health Problems (ICD-10) diseases, a medical classification list from the World Health Organization [14]. The IP can list up to three disorders during the medical disability assessment. These diagnoses are divided into 14 categories, according to the ICD-10 classification, which we used in this study. .

### Comorbidity

For this study we created a comorbidity classification scheme based on the CAS as established classification schemes did not fit our study data. CAS includes only information about the existence of disorders, and not about their severity. The IP lists in CAS the first (main) diagnosis for which an individual claims inability to work, and possibly a second and third diagnoses. The IP will only mention a second or third diagnoses if he or she believes that these result in important, additional functional disabilities. Therefore, in the present study, we considered all second and third diagnoses as comorbidity, independent of the disease categories of the ICD-10 classification that the disorders belong to.

We defined comorbidity as two or three disorders being listed for an individual. To gain insight into the disorders present in cases of comorbidity, we divided the diagnostic categories into mental (mental disorders) and physical disorders (all remaining disorders). Possible conditions of comorbidity were multiple mental disorders, multiple physical disorders or a comorbidity of mental and physical disorders.

### Continuing eligibility for disability benefit

We used a follow-up period of five years. For each individual in the study sample, we used SSI registration data to determine whether the benefit ended within one, three or five years after the date of approval (and if so, for what reason). During this five-year follow-up period there were no major changes in legislation or working processes that could have influenced our results.

### Statistical analysis

Statistical analyses were performed in RStudio for Windows, version 0.99.902. The chi-square test for categorical variables was used to compare socio-demographic characteristics, disorders, comorbidity and outflow from disability benefits among various groups of individuals. Multinomial regression models were used to test for relationships between disorders, comorbidity and outflow from disability benefit respectively (dependent variable) and socio-demographic characteristics, disorders and comorbidity (independent variables) while taking confounding effects into account. The level of significance was set at *p* < 0.05.

## Results

### Characteristics of the study population

The socio-demographic characteristics and disorders of the study population are summarized in Table 2. To facilitate interpretation, all numbers were rounded to the nearest ten. The mean age was 46.8 years (SD, 10.6) and the number of men and women was approximately equal.

**Table 2:** Summary of socio-demographic characteristics and disorders

	Study sample n = 31,730 n (%)
Socio-demographics	
Gender	
Male	15,650 (49)
Female	16,090 (51)
Age category	
< 35	5210 (16)
35–44	7060 (22)
45–54	10,160 (32)
55+	9300 (29)
Educational level	
Low	16,820 (53)
Secondary	7390 (23)
High	3500 (11)
Unknown	4040 (13)
Disorders	
Main causes of work disability	
Cancer	2510 (8)
Cardiovascular	2790 (9)
Mental	10,870 (34)
Musculoskeletal	8410 (27)
Nervous system	2090 (9)
Other	4260 (13)

Table 3 shows the age categories and educational levels divided by gender. Women who qualified for disability benefits were on average younger [χ^2^ (df = 3; *n* = 31,733) = 519.33, *p* = 0.000], and more highly educated [χ^2^ (df = 2; *n* = 27,697) = 262.43, *p* = 0.000] than men.

**Table 3:** Age and educational level by gender

	Gender n (%)	
	Male	Female
Age category		
< 35	2070 (13)	3140 (20)
35–44	3230 (21)	3830 (24)
45–54	4940 (32)	5220 (33)
55+	5400 (35)	3890 (24)
Educational level		
Low	8860 (57)	7960 (50)
Secondary	3290 (21)	4100 (26)
High	1400 (9)	2100 (13)
Unknown	2100 (13)	1940 (12)

### Main diagnoses for work disability

Table 2 shows the main disorders as listed by the IP for medical disability assessment. Mental disorders were most often mentioned, followed by musculoskeletal disorders, nervous system disorders, cancer and cardiovascular system disorders. The category “other” consisted of various classes of physical disorders that were listed less frequently (among others respiratory system, digestive system and genitourinary system disorders).

Table 4 shows that the main diagnosis for work disability differed significantly among age categories [χ^2^ (df = 15; *n* = 31,733) = 3306.1, *p* = 0.000], with mental disorders as the main diagnosis for individuals younger than 55 years, and musculoskeletal disorders the main diagnosis for individuals 55 years and older. The differences in leading diagnoses between men and women were statistically significant but smaller [χ^2^ (df = 5; n = 31,733) = 541.15, *p* = 0.000]. Mental and musculoskeletal disorders were registered with approximately the same frequency for women and men. However, cancer was more often registered for women (mostly breast cancer) and cardiovascular disorders for men (mostly stroke, heart attack).

**Table 4:** Main diagnosis by age, gender and educational level

	Main diagnosis n (%)					
	Mental	Musculoskeletal	Nervous system	Cardiovascular	Cancer	Other
Gender						
Male	5160 (33)	4170 (27)	1460 (9)	1860 (12)	890 (6)	2110 (13)
Female	5710 (35)	4240 (26)	1450 (9)	930 (6)	1620 (10)	2150 (13)
Age category						
< 35	2970 (57)	1000 (19)	460 (9)	80 (1)	120 (2)	590 (11)
35–44	3150 (45)	1690 (24)	670 (10)	320 (5)	360 (5)	860 (12)
45–54	3010 (30)	2910 (29)	950 (9)	1010 (10)	960 (9)	1340 (13)
55+	1740 (19)	2810 (30)	820 (9)	1380 (15)	1070 (12)	1470 (16)
Educational level						
Low	5350 (32)	5860 (35)	1210 (7)	1470 (9)	830 (5)	2100 (12)
Secondary	2680 (36)	1820 (25)	750 (10)	610 (8)	470 (6)	1070 (14)
High	1510 (43)	470 (13)	450 (13)	280 (8)	280 (8)	500 (14)

The leading diagnosis also differed for educational level [χ^2^ (df = 10; *n* = 27,697) = 857.12, p = 0.000]. Individuals who were more highly educated suffered more often from mental disorders, nervous system disorders and cancer, whereas individuals who were less educated suffered more often from musculoskeletal and cardiovascular disorders.

### Comorbidity

More than half of the individuals in the study population (55.8%) suffered from comorbidity. Table 5 shows that work disability due to comorbidity was mentioned as frequently for men as for women [χ^2^ (df = 1; *n* = 31,733) = 0.356, *p* = 0.551], and more often for older [χ^2^ (df = 3; n = 31,733) = 92.866, *p* = 0.000] and less educated individuals [χ^2^ (df = 2; *n* = 27,697) = 168.65, *p* = 0.000]. Considering the main diagnoses for work disability [χ^2^ (df = 5; n = 31,733) = 765.29, *p* = 0.000], individuals with cancer suffered least often from comorbidity and individuals with musculoskeletal disorders most often.

**Table 5:** Comorbidity by age, gender, educational level and main diagnosis

	Comorbidity n (%)	
	Yes	No
Gender		
Male	8700 (56)	6940 (44)
Female	9010 (56)	7080 (44)
Age category		
< 35	2690 (52)	2520 (48)
35–44	3770 (53)	3290 (47)
45–54	5780 (57)	4390 (43)
55+	5470 (59)	3830 (41)
Educational level		
Low	10,240 (61)	6580 (39)
Secondary	4060 (55)	3330 (45)
High	1760 (50)	1730 (50)
Main diagnosis causing work disability		
Cancer	930 (37)	1580 (63)
Cardiovascular	1640 (59)	1150 (41)
Mental	6310 (58)	4560 (42)
Musculoskeletal	5180 (62)	3230 (38)
Nervous system	1190 (41)	1710 (59)
Other	2460 (58)	1800 (41)

**Fig. 1:**
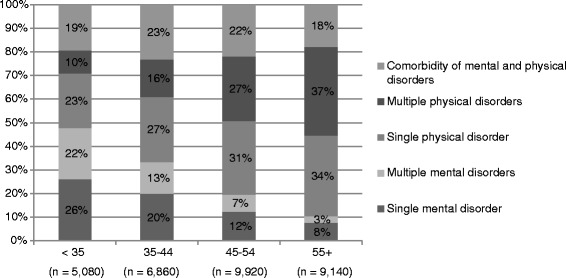
Comorbidity for individuals of various age groups

Figure 1 shows the (combination of types of) diagnoses registered by the IP during a medical disability assessment for each age category. For younger individuals, comorbidity was most often a combination of multiple mental disorders, and for older individuals it was most often a combination of multiple physical diagnoses (musculoskeletal, nervous or cardiovascular disorders).

### Continuing eligibility for disability benefits

Of the individuals who had been granted a disability benefit in the study time frame, 964 individuals (3%) left in the first year, 2607 (8%) in the second and third year, and 2258 (7%) in the fourth and fifth year. All other individuals (25,907; 82%) continued to receive disability benefits after five years. Outflow was caused by retirement (37%), death (30%), improvement of the medical condition (such that individuals could earn at least 65% of former wages [28%]), or other reasons such as imprisonment or pregnancy (5%).

Table 6 shows that the main reason for outflow in the first year was death, in the second and third year income loss lower than 35% of former wages earned, and in the fourth and fifth year retirement. Table 7 shows that the differences for gender [χ^2^ (df = 3; *n* = 31,733) = 37.549, *p* = 0.000] and age categories [χ^2^ (df = 9; n = 31,733) = 2259.8, *p* = 0.000] were statistically significant, but small. There was no difference for education categories [χ^2^ (df = 6; *n* = 27,697) = 5.9007, *p* = 0.434].

**Table 6:** Timing and reason for outflow of disability benefit

	Reason for outflow of disability benefit n (%)				
	Retirement	Death	Income loss < 35%	Other	Total
Outflow of disability benefit					
1st year	200 (21)	490 (52)	170 (18)	100 (10)	960 (100)
2nd or 3rd year	790 (30)	750 (29)	930 (36)	140 (5)	2610 (100)
4th or 5th year	1140 (50)	510 (22)	540 (24)	80 (3)	2260 (100)

**Table 7:** Outflow of disability benefits by age, gender, education, main diagnosis and comorbidity

	Outflow of disability benefit n (%)			
	1st year	2nd or 3rd year	4th or 5th year	Continuing eligibility
Gender				
Male	510 (3)	1330 (8)	1230 (8)	12,590 (80)
Female	450 (3)	1280 (8)	1030 (6)	13,330 (83)
Age category				
< 35	110 (2)	370 (7)	200 (4)	4530 (87)
35–44	130 (2)	510 (7)	220 (3)	6200 (88)
45–54	230 (2)	560 (5)	380 (4)	9000 (88)
55+	490 (5)	1170 (13)	1450 (16)	6180 (66)
Education level				
Low	360 (2)	1300 (8)	1200 (7)	13,950 (83)
Secondary	160 (2)	570 (8)	450 (7)	6150 (83)
High	70 (2)	290 (8)	270 (8)	2860 (82)
Main diagnosis causing work disability				
Mental	160 (1)	670 (6)	540 (5)	9500 (87)
Musculoskeletal	190 (2)	650 (8)	570 (7)	7000 (83)
Nervous system	50 (2)	150 (5)	220 (7)	2490 (86)
Cardiovascular	60 (2)	250 (9)	300 (11)	2180 (78)
Cancer	370 (15)	490 (19)	250 (10)	1410 (56)
Other	140 (3)	400 (9)	390 (9)	3330 (78)
Comorbidity				
Single mental disorder	80 (2)	310 (7)	220 (5)	4020 (87)
Multiple mental disorders	30 (1)	160 (5)	130 (4)	2680 (89)
Single physical disorder	490 (5)	940 (10)	770 (8)	7070 (76)
Multiple physical disorders	250 (3)	740 (10)	710 (9)	6010 (78)
Mental and physical disorders	100 (2)	410 (6)	390 (6)	5490 (86)

The outflow of disability benefits differed by class of the leading diagnoses for work disability [χ^2^ (df = 15; *n* = 31,733) = 2148.4, *p* = 0.000]. In the first year, the outflow consisted mainly of individuals diagnosed with cancer who died within one year after their disability benefit was approved. After four and five years, more older individuals with musculoskeletal and cardiovascular disorders left because of retirement.

Continuing eligibility for disability benefits was highest for individuals with single or multiple mental disorders and individuals facing a comorbidity of mental and physical disorders, and lowest for individuals with single or multiple physical disorders [χ^2^ (df = 15; n = 31,733) = 653.9, *p* = 0.000].

## Discussion

### Main findings

Disability diagnoses differed significantly among age groups and education categories; mental disorders were the main diagnosis for work disability for younger and more highly educated individuals, and physical disorders (mainly musculoskeletal, cardiovascular and cancer) for older and less educated individuals. The differences between men and women were small.

Multiple diagnoses were registered for more than half of the population. Older and less educated individuals suffered relatively often from comorbidity.

In the five-year follow-up, the continuation of disability benefits for five years or more after approval was high. Only 18% of the individuals in our study sample discontinued their disability benefits in the five-year follow-up period. Continuing eligibility for disability benefit was highest for individuals with (multiple) mental disorders and those with a comorbidity of mental and physical disorders, and lowest for individuals with (multiple) physical disorders.

### Interpretation of findings and comparison with other studies

The current finding that women who qualify for disability benefits are on average younger and more educated than men confirms previously reported findings. A reason for this difference in age of entry to disability benefits is the relatively low number of older women among the insured population [15]. This is most likely because a few decades ago many women did not continue paid work after giving birth. More recently, the proportion of women aged 50–64 years in the workforce has increased, and is still increasing such that the employment gap between men and women is becoming smaller [16, 17].

We found that mental disorders were the main diagnosis for work disability. This is in line with the finding that mental disorders are the leading cause of sickness absence and work disability in OECD countries [18]. Research shows that mental health impairments have increased over the past years. This could be explained by the changing content of communication and social networks, and the changed and increased job demands in the workplace [19, 20]. All these factors make it increasingly difficult for workers with mental health problems to return to work.

Our finding that younger individuals in particular suffer from mental disorders corresponds with the finding that younger generations are at increased risk for mental health problems [1, 21]. Two major explanations are changes in the workplace that have increased the prevalence of work-related stress, and the changing content of communication and social networks. Our finding is a problem because work absence due to mental illness is often long lasting. In the Netherlands, the median duration of absence due to mental illness has increased. The probability of resuming work decreases with the increasing duration of absence due to illness [22]. Conversely, the prevalence of musculoskeletal disorders as the main diagnosis for work disability is higher for older individuals. An association between age and musculoskeletal disorders is generally found in several studies [23, 24].

Concerning the relationship between the main diagnoses for work disability and education, we found that individuals who were more highly educated suffered more often from mental disorders, nervous system disorders and cancer, and individuals who were less educated suffered more often from musculoskeletal and cardiovascular disorders. This may be due to differences in the type of jobs and workplaces for these two groups.

A considerable part of our study population (65%) suffered from comorbidity. Research shows the importance of comorbidity as a predictor for long-term work disability [25]. Multiple physical symptoms have a generic negative influence on the effectiveness of treatment for symptoms of depression and anxiety in primary care [26].

The duration of disability benefits is longer for older workers, when the main diagnosis for work disability is a mental disorder and when comorbidity is present, and only related to gender and education to a limited extent. Similar findings can be found in the literature on prognostic factors for long-term disability due to mental disorders [27]. Of the individuals in the study population, 82% had continuing eligibility for their disability benefits five years after approval. An application for disability benefits can be requested after two years of sick leave. This means that individuals who qualify for disability benefits have already been sick for a long period of time and have severe disorders that may be more difficult to treat. In addition, in these two years, the system does not offer many incentives for individuals to return to work. Hence, (partial) recovery after two years of sick leave would be unexpected. This could explain the low outflow in the present cohort.

### Strengths and limitations of the study

An important strength of this study is the large study sample. By covering the entire Dutch population applying for long-term disability benefits, with a one-year inflow period and a five-year follow-up period, our study population is highly inclusive. To our knowledge studies about individuals at risk for long-term disability benefits generally focus on one specific diagnosis, while we included all individuals who were granted a disability benefit in the Netherlands in the one-year inflow period. By doing this, we can give an overview of all diagnoses for which individuals claim work disability. In addition, in most studies in the field of work disability the follow-up period is only one or two years, while we were able to use a follow-up period of five years after approval of the benefit.

We performed a similar study with individuals who were granted a disability benefit in 2015 and the individual characteristics, main diagnoses for work disability and comorbidity numbers were similar to the ones in this study, thus confirming our results here. The figures on the socio-demographic characteristics of individuals receiving disability benefits are also consistent with SSI data [28].

A limitation of only testing for bivariate relationships is that it is not possible to control for confounding effects. Therefore, we have also performed three multinomial regression analyses (with main diagnosis for work disability, comorbidity and continuing eligibility for disability benefits respectively as the dependent variables). The results of these regression analyses can be found in Appendix A. They confirm the statistical bivariate relations that we found with the chi-square tests.

A study limitation is that data was not collected for research purposes, but rather registered by SSI employees for administration purposes. Although careful registration is important for internal processes, employees might not have been fully aware of the importance of complete and comprehensive administration and some records contained missing data. For that reason, we had to exclude 4036 individuals from our analyses concerning the education level as their values were missing. In this study, we considered only socio-demographic factors, main diagnosis, comorbidity and claim duration. However, there could be other factors (partly) explaining our findings.

### Practical implications

This study provides insight into the socio-demographic factors and health complaints of individuals who qualify for disability benefits in the Netherlands and shows that continuing eligibility for disability benefits is high. This information can help identify specific at-risk groups when policies are aimed at decreasing the number of applications for disability benefits. The results of this study may be useful when policy makers investigate how to reduce long-term disability benefits. In this context, the main focus should be on individuals who leave for reasons other than retirement and death. Increased understanding of the characteristics of this group and how to support them in returning to work is needed. Conducting re-assessments, wherein the SSI would assess whether or not someone’s health had improved enough so that their earning capacity had increased, is a possible way to motivate individuals to return to work.

## Conclusions

This study provides an overview of the socio-demographic characteristics and diagnoses of individuals who have been granted a disability benefit, and examines the duration of their benefit. Therefore, it contributes to insight into the range of diagnoses and how they differ in age, gender and education. An understanding of factors associated with long-term work disability may be helpful to identify groups of individuals who are at risk for continuing eligibility for disability benefits and to develop effective interventions for these groups.

## Appendix 1

**Table 8:** Relation between main diagnosis for work disability and socio-demographic characteristics

Main diagnosis^a^	Independent variables	Coefficient	Standard error	Z statistic	*p*-value	Relative risk ratio
Musculoskeletal	Intercept	-2.484	0.074	−33.47	0.000	
	Gender					
	Male	1.00 (ref)				
	Female	0.095	0.031	3.10	0.002	1.10
	Age	0.055	0.001	37.17	0.000	1.06
	Educational level					
	Low	1.00 (ref)				
	Secondary	−0.379	0.039	−10.27	0.000	0.68
	High	−1.293	0.057	−22.59	0.000	0.27
	Unknown	−1.820	0.072	−25.41	0.000	0.16
Nervous system	Intercept	-3.135	0.104	−30.15	0.000	
	Gender					
	Male	1.00 (ref)				
	Female	−0.018	0.043	−0.43	0.666	0.98
	Age	0.037	0.002	28.23	0.000	1.04
	Educational level					
	Low	1.00 (ref)				
	Secondary	0.284	0.053	5.37	0.000	1.33
	High	0.260	0.063	4.11	0.000	1.30
	Unknown	0.447	0.062	7.21	0.000	1.56
Cardiovascular system	Intercept	-6.185	0.141	−43.97	0.000	
	Gender					
	Male	1.00 (ref)				
	Female	−0.518	0.046	−11.17	0.000	0.60
	Age	0.106	0.003	40.37	0.000	1.11
	Educational level					
	Low	1.00 (ref)				
	Secondary	0.014	0.056	0.25	0.805	1.01
	High	−0.395	0.074	−5.33	0.000	0.67
	Unknown	−0.001	0.066	−0.02	0.985	1.00
Cancer	Intercept	−6.615	0.144	−45.87	0.000	
	Gender					
	Male	1.00 (ref)				
	Female	0.775	0.049	15.92	0.000	2.17
	Age	0.091	0.003	34.93	0.000	1.10
	Educational level					
	Low	1.00 (ref)				
	Secondary	0.257	0.064	4.00	0.000	1.29
	High	0.096	0.077	1.28	0.200	1.10
	Unknown	1.396	0.059	23.52	0.000	4.04
Other	Intercept	−3.303	0.093	−35.48	0.000	
	Gender					
	Male	1.00 (ref)				
	Female	0.061	0.037	1.63	0.103	1.06
	Age	0.051	0.002	28.23	0.000	1.05
	Educational level					
	Low	1.00 (ref)				
	Secondary	0.107	0.045	2.36	0.018	1.11
	High	−0.197	0.059	−3.36	0.000	0.82
	Unknown	0.035	0.057	0.61	0.543	1.04

^a^Reference category is mental disorders

**Table 9:** Relation between comorbidity and socio-demographic characteristics and main diagnosis for work disability

Comorbidity^a^	Independent variables	Coefficient	Standard error	Z statistic	*p*-value	Relative risk ratio
Multiple mental, multiple physical or comorbidity of mental and physical disorders	Intercept	-0.231	0.057	−4.08	0.000	
	Gender					
	Male	1.00 (ref)				
	Female	0.103	0.024	4.37	0.000	0.80
	Age	0.016	0.001	13.99	0.000	1.11
	Educational level					
	Low	1.00 (ref)				
	Secondary	−0.193	0.029	−6.71	0.000	0.82
	High	−0.382	0.038	−9.96	0.000	0.68
	Unknown	−0.670	0.037	−17.88	0.000	0.51
	Main diagnosis					
	Mental	1.00 (ref)				
	Musculoskeletal	−0.044	0.031	−1.40	0.160	0.96
	Nervous system	−0.723	0.043	−16.73	0.000	0.49
	Cardiovascular	−0.121	0.045	−2.66	0.008	0.89
	Cancer	−0.887	0.048	−18.41	0.000	0.41
	Other	−0.095	0.038	−2.53	0.013	0.91

^a^Reference category is no comorbid disorders (single mental or single physical disorder)

**Table 10:** Relation between continuing eligibility for disability benefit and socio-demographic characteristics, main diagnosis for work disability and comorbidity

Outflow of disability benefit^a^	Independent variables	Coefficient	Standard error	Z statistic	*p*-value	Relative risk ratio
Outflow in 1st year	Intercept	-5.934	0.230	−25.78	0.000	
	Gender					
	Male	1.00 (ref)				
	Female	−0.255	0.070	−3.64	0.000	0.78
	Age	0.042	0.004	10.36	0.000	1.04
	Educational level					
	Low	1.00 (ref)				
	Secondary	0.057	0.099	0.58	0.564	1.06
	High	−0.024	0.133	−0.18	0.860	0.98
	Unknown	1.033	0.087	11.84	0.000	2.81
	Main diagnosis					
	Mental	1.00 (ref)				
	Musculoskeletal	−0.347	0.214	−1.62	0.105	0.71
	Nervous system	−0.925	0.249	−3.71	0.000	0.40
	Cardiovascular	−0.761	0.241	−3.15	0.002	0.47
	Cancer	1.351	0.213	6.33	0.000	3.86
	Other	−0.079	0.221	−0.17	0.864	0.96
	Comorbidity					
	Single mental disorder	1.00 (ref)				
	Multiple mental disorders	−0.302	0.211	−1.43	0.153	0.74
	Single physical disorder	0.876	0.242	3.62	0.000	2.10
	Multiple physical disorders	0.617	0.246	2.51	0.012	1.85
	Mental and physical	−0.066	0.184	−0.36	0.721	0.94
Outflow in 2nd or 3rd year	Intercept	−4.060	0.126	− 32.15	0.000	
	Gender					
	Male	1.00 (ref)				
	Female	−0.079	0.043	−1.83	0.067	0.92
	Age	0.034	0.002	14.57	0.000	1.03
	Educational level					
	Low	1.00 (ref)				
	Secondary	0.068	0.054	1.27	0.203	1.07
	High	0.088	0.070	1.25	0.210	1.09
	Unknown	0.204	0.063	3.21	0.001	1.22
	Main diagnosis					
	Mental	1.00 (ref)				
	Musculoskeletal	−0.114	0.108	−1.62	0.293	0.89
	Nervous system	−0.572	0.133	−4.32	0.000	0.56
	Cardiovascular	−0.098	0.122	−0.81	0.421	0.91
	Cancer	1.029	0.116	8.85	0.000	2.80
	Other	0.184	0.115	1.60	0.110	1.20
	Comorbidity					
	Single mental disorder	1.00 (ref)				
	Multiple mental disorders	−0.133	0.100	−1.32	0.186	0.88
	Single physical disorder	0.238	0.125	1.90	0.058	1.27
	Multiple physical disorders	0.171	0.127	1.35	0.178	1.19
	Mental and physical	−0.180	0.095	−1.89	0.880	0.84
Outflow in 4th or 5th year	Intercept	−6.879	0.170	−40.54	0.000	
	Gender					
	Male	1.00 (ref)				
	Female	−0.048	0.046	−1.04	0.300	0.95
	Age	0.087	0.003	28.97	0.000	1.09
	Educational level					
	Low	1.00 (ref)				
	Secondary	0.086	0.057	1.50	0.133	1.09
	High	0.103	0.073	1.42	0.157	1.11
	Unknown	−0.142	0.074	−1.92	0.055	0.87
	Main diagnosis					
	Mental	1.00 (ref)				
	Musculoskeletal	−0.254	0.112	−2.27	0.023	0.78
	Nervous system	−0.102	0.127	−0.80	0.422	0.90
	Cardiovascular	−0.035	0.122	−0.29	0.772	0.97
	Cancer	0.371	0.127	2.92	0.004	1.45
	Other	0.193	0.118	1.64	0.101	1.21
	Comorbidity					
	Single mental disorder	1.00 (ref)				
	Multiple mental disorders	0.073	0.117	0.62	0.535	1.08
	Single physical disorder	0.295	0.134	2.20	0.028	1.34
	Multiple physical disorders	0.195	0.135	1.44	0.150	1.21
	Mental and physical	−0.016	0.104	−0.15	0.880	0.98

^a^Reference category is continuing eligibility for disability benefit

## Abbreviations

CAS: Dutch Classification of Occupational Health and Social Insurance
ICD-10: International Statistical Classification of Disease and Related Health Problems
IP: Insurance physician
SSI: Social Security Institute
WIA: Dutch Work and Income Act
